# Supplementary material for: Wind prevents cliff-breeding birds from accessing nests through loss of flight control
Source: eLife. 2019 Jun 12;8:e43842. doi: 10.7554/eLife.43842 (PMC6561702; doi:10.7554/eLife.43842)
Supplement: Supplementary file 1. — Numbers of guillemots (GM) are taken from the 2015 Skomer Island breeding bird survey (Stubbings et al., 2015). [file elife-43842-supp1.docx]

**Supplementary file 1.** Summary information for the breeding cliffs where landing data were collected from April - July 2016, and April - May 2017. Numbers of guillemots (GM) are taken from the 2015 Skomer Island breeding bird survey (19).

| Colony | GM | Colony centre | Anemometer location | Cliff  height (m) | Topography | Orientation |
| --- | --- | --- | --- | --- | --- | --- |
| North Haven | 723 | 51.737602  -5.278370 | 51.441466  -5.165364 | 35 | Open bay | 318° |
| High Cliff | 2182 | 51.731549  -5.288387 | 51.435676  -5.172086 | 56 | Open bay | 46° |
| Mew Stone | 315 | 51.727822  -5.291966 | 51.434391  -5.173042 | 60 | Sheltered islet | 352° |
| The Wick | 4304 | 51.730731  -5.296035 | 51.435391  -5.174458 | 73 | Sheltered bay | 343° |
| Bull Hole | 5771 | 51.740416  -5.309871 | 51.442751  -5.182546 | 68 | Partially sheltered bay | 355° |
